# Exercise programs for people with dementia

**DOI:** 10.1590/1516-3180.20141323T2

**Published:** 2014-04-14

**Authors:** Dorothy Forbes, Emily J. Thiessen, Catherine M. Blake, Scott S. Forbes, Sean Forbes

## Abstract

**BACKGROUND:**

This is an update of our previous 2008 review. Several recent trials and systematic reviews of the impact of exercise on people with dementia are reporting promising ﬁndings.

**OBJECTIVE:**

*Primary:* Do exercise programs for older people with dementia improve cognition, activities of daily living (ADLs), challenging behaviour, depression, and mortality in older people with dementia? *Secondary:* Do exercise programs for older people with dementia have an indirect impact on family caregivers’ burden, quality of life, and mortality? Do exercise programs for older people with dementia reduce the use of healthcare services (e.g. visits to the emergency department) by participants and their family caregivers?

**METHODS:**

*Search methods:* We identiﬁed trials for inclusion in the review by searching ALOIS (www.medicine.ox.ac.uk/alois), the Cochrane Dementia and Cognitive Improvement Group’s Specialised Register, on 4 September 2011, and again on 13 August 2012. The search terms used were: ’physical activity’ OR exercise OR cycling OR swim* OR gym* OR walk* OR danc* OR yoga OR ‘tai chi’.

*Selection criteria:* In this review, we included randomized controlled trials in which older people, diagnosed with dementia, were allocated either to exercise programs or to control groups (usual care or social contact/activities) with the aim of improving cognition, ADLs, behaviour, depression, and mortality. Secondary outcomes related to the family caregiver(s) and included caregiver burden, quality of life, mortality, and use of healthcare services.

*Data collection and analysis:* Independently, at least two authors assessed the retrieved articles for inclusion, assessed methodological quality, and extracted data. Data were analysed for summary effects using RevMan 5.1 software. We calculated mean differences or standardized mean difference (SMD) for continuous data, and synthesized data for each outcome using a ﬁxed-effect model, unless there was substantial heterogeneity between studies, when we used a random-effects model. We planned to explore heterogeneity in relation to severity and type of dementia, and type, frequency, and duration of exercise program. We also evaluated adverse events.

**MAIN RESULTS:**

Sixteen trials with 937 participants met the inclusion criteria. However, the required data from three trials and some of the data from a fourth trial were not published and not made available. The included trials were highly heterogeneous in terms of subtype and severity of participants’ dementia, and type, duration and frequency of exercise. Only two trials included participants living at home. Our meta-analysis suggested that exercise programs might have a signiﬁcant impact on improving cognitive functioning (eight trials, 329 participants; SMD 0.55, 95% conﬁdence interval (CI) 0.02 to 1.09). However, there was substantial heterogeneity between trials (I 2 value 80%), most of which we were unable to explain. We repeated the analysis omitting one trial, an outlier, that included only participants with moderate or severe dementia. This reduced the heterogeneity somewhat (I2 value 68%), and produced a result that was no longer signiﬁcant (seven trials, 308 participants; SMD 0.31, 95% CI -0.11 to 0.74). We found a signiﬁcant effect of exercise programs on the ability of people with dementia to perform ADLs (six studies, 289 participants; SMD 0.68, 95% CI 0.08 to 1.27). However, again we observed considerable unexplained statistical heterogeneity (I2 value 77%) in this meta-analysis. This means that there is a need for caution in interpreting these ﬁndings. In further analyses, we found that the burden experienced by informal caregivers providing care in the home may be reduced when they supervise the participation of the family member with dementia in an exercise program (one study, 40 participants; MD -15.30, 95% CI -24.73 to -5.87), but we found no signiﬁcant effect of exercise on challenging behaviours (one study, 110 participants; MD -0.60, 95% CI -4.22 to 3.02), or depression (six studies, 341 participants; MD -0.14, 95% CI -0.36 to 0.07). We could not examine the remaining outcomes, quality of life, mortality, and healthcare costs, as either the appropriate data were not reported, or we did not retrieve trials that examined these outcomes.

**AUTHORS’ CONCLUSIONS:**

There is promising evidence that exercise programs can have a signiﬁcant impact in improving ability to perform ADLs and possibly in improving cognition in people with dementia, although some caution is advised in interpreting these ﬁndings. The programs revealed no signiﬁcant effect on challenging behaviours or depression. There was little or no evidence regarding the remaining outcomes of interest.

**This is the abstract of a Cochrane Review published in the Cochrane Database of Systematic Reviews (CDSR) 2013, issue 12, Art. No.: CD006489. DOI:** 10.1002/14651858.CD006489.pub3 (http://onlinelibrary.wiley.com/doi/10.1002/14651858.CD006489.pub3/abstract;jsessionid=EB059310003BD3AC29CC6D77710D8779.f01t02). For full citation and authors’ details see reference 1.

**The full text is available from:** http://onlinelibrary.wiley.com/doi/10.1002/14651858.CD006489.pub3/abstract.

**The abstract is also available in the Portuguese, French and Spanish languages from:** http://summaries.cochrane.org/CD006489/exercise-programs-for-people-with-dementia.
